# Genome wide characterization of barley NAC transcription factors enables the identification of grain-specific transcription factors exclusive for the Poaceae family of monocotyledonous plants

**DOI:** 10.1371/journal.pone.0209769

**Published:** 2018-12-28

**Authors:** Emiko Murozuka, Julio A. Massange-Sánchez, Kasper Nielsen, Per L. Gregersen, Ilka Braumann

**Affiliations:** 1 Carlsberg Research Laboratory, Copenhagen, Denmark; 2 Department of Molecular Biology and Genetics, Aarhus University, Slagelse, Denmark; Ben-Gurion University, ISRAEL

## Abstract

The plant NAC transcription factors depict one of the largest plant transcription factor families. They regulate a wide range of different developmental processes and most probably played an important role in the evolutionary diversification of plants. This makes comparative studies of the NAC transcription factor family between individual species and genera highly relevant and such studies have in recent years been greatly facilitated by the increasing number of fully sequenced complex plant genomes. This study combines the characterization of the NAC transcription factors in the recently sequenced genome of the cereal crop barley with expression analysis and a comprehensive phylogenetic characterization of the NAC transcription factors in other monocotyledonous plant species. Our results provide evidence for the emergence of a NAC transcription factor subclade that is exclusively expressed in the grains of the Poaceae family of grasses. These notably comprise a number of cereal crops other than barley, such as wheat, rice, maize or millet, which are all cultivated for their starchy edible grains. Apparently, the grain specific subclade emerged from a well described subgroup of NAC transcription factors associated with the senescence process. A promoter exchange subsequently resulted in grain specific expression. We propose to designate this transcription factor subclade Grain-NACs and we discuss their involvement in programmed cell death as well as their potential role in the evolution of the Poaceae grain, which doubtlessly is of central importance for human nutrition.

## Introduction

The development of a plant from the germinating seed to the mature plant setting seeds of its own is governed by the activity of a vast number of different transcription factors (TFs). In higher plants 58 different TF gene families are known, comprising from only a few to, in rare cases, more than 200 genes [1]. One of the largest TF families depict the plant specific NAC (NAM-ATAF1/2-CUC2) TFs, with often more than 100 members in both monocot and dicot species [2–11].

NAC TFs are defined through their highly conserved N-terminal part, the NAC domain, which consists of five sub-domains, designated A to E [4]. The E sub-domain can, however, be missing. The NAC domain mediates homo- or heterodimerization, a requirement for the DNA binding properties of the NAC TFs [12]. The C-terminal part of the protein by contrast is in general highly divergent, both in length and structure. It, however, contains short motifs conserved within subfamilies, which are believed to be of importance for the trans-activating function of the NAC TFs [13,14].

NAC TFs are involved in the regulation of a range of different developmental processes in plants, of which two have particularly been in focus: Secondary cell wall formation during development of vascular tissues (reviewed by [15]), and the senescence and nutrient remobilization processes taking place in vegetative tissues prior to whole plant senescence in monocarpic plants (reviewed by [16]). In addition, a plethora of reports on the association of NAC TFs with both abiotic and biotic stress responses in plants is available (reviewed by [17]). One striking common denominator observed across the developmental processes associated with NAC TFs, is the occurrence of programmed cell death (PCD), e.g. during the formation of tracheary elements and, rather obviously, during senescence processes [18].

Phylogenetic relationships of the NAC TF family across plant species have been described in several reports [4,8,10,14,19]. These show the occurrence of lineage specific expansions and sometimes extinctions of certain subfamilies [19], suggesting that NAC TFs may have played an important role in the evolutionary diversification of plants. It has, for instance, been hypothesized that the group of NAC TFs involved in formation of water conducting xylem vessels depict the basis for the development of land plants [20]. This example illustrates that it is relevant to characterize the NAC TF family at species or genera level to observe evolutionary specializations. This is certainly facilitated by the continuously increasing number of fully sequenced even complex plant genomes, wheat and barley being recent examples [21,22]. The characterization of the NAC TF genes encoded in the barley genome, which we report in this study, prompted us to investigate a specific specialization event, the emergence of a NAC TF subclade only present in the Poaceae family of monocotyledonous plants. These TFs are strongly and exclusively expressed in the specialized fruit of the Poaceae, the caryopsis, commonly referred to as grain. Hence, we designate these TFs as Grain-NACs. Notably, the Poaceae family comprises a large number of cereal crops of particular importance for human nutrition, such as rice, wheat, maize and barley, which are cultivated for their starchy edible grains. We will further discuss the association of the Grain-NACs with another process associated with the expression of NAC TFs: the PCD of the endosperm occurring during grain maturation. Based on the hypothesis that NAC TFs have played an important role in evolutionary diversification of plants, we propose that the emergence of Grain-NAC TFs in the Poaceae was involved in the evolution of the cereal grain.

## Material and methods

### Identification of barley NAC TFs

The recently published new barley genome assembly, along with annotations and 333,571 protein sequences (for all gene models) [22], was used to identify barley NAC (HvNAC) TFs. HvNACs were identified by (1) an annotation search of the human readable description available for the barley gene models using the keywords “NAC”, “NAM” and “PF02365”, (2) a BLASTp [23] search using all known protein sequences from rice (*Oryza sativa* ‘Japonica’), *Brachypodium distachyon*, *Arabidopsis thaliana* NAC TFs as well as the barley NAC TFs, which had been identified by the annotation search as query, (3) a HMMER [24] search of the profile hidden Markov model for PF02365 (extracted from the Pfam database release 30 [25]) with hmmsearch version 3.1b3 (http://hmmer.org/) run against all 333,571 barley protein sequences with all heuristics turned off and using the gathering bit scores in the model as thresholds and (4) tBLASTn (evalue cutoff: 1e-25) using the sequences from the HvNACs, which had already been identified by the attempts previously listed as query [23,26]. The protein sequences from rice, *B*. *distachyon* and *A*. *thaliana* NAC TFs were collected from publicly available databases and publications [6,9,27–33].

The presence of the NAM domain PF02365 was verified in all identified NAC TFs with hmmsearch and peptide sequences for the domains were extracted. Some proteins had more than one NAC domain and in such cases, they were all kept. Sequences that could not be verified, were excluded. For the tBLASTn hits, flanking sequences of 5kb were added, and EMBOSS getorf v. 6.5.7.0 [34] was used to predict open reading frames used as input for hmmsearch.

When several protein sequences (corresponding to different gene models) were identified for one gene locus, the most complete one was manually selected, based on whether the gene model comprised a start codon and on sequence length (the longest sequence was preferred). Some identified protein sequences however still appeared incomplete, possibly due to inaccurate gene prediction or sequence gaps. In such cases, the sequences were used as query for a BLASTp search against the database of predicted proteins from the barley WGS Morex Assembly v3 [35] to retrieve full length sequences where possible.

DLN and LVFY motifs in the NAC domain sequences were identified by motif search applying default settings in CLC Main Workbench 8.0 (QIAGEN Aarhus, Denmark; https://www.qiagenbioinformatics.com/).

### Identification of segmental and tandem duplications

Segmental or tandem duplications were identified using BLASTp with full protein sequences and BLASTn with coding sequences as queries on the IPK Barley BLAST Server (http://webblast.ipk-gatersleben.de/barley_ibsc/) with the default settings. Sequence pairs with more than 90% identity were considered as duplicated sequences. In some cases, it was not possible to evaluate duplication events due to incomplete sequences. Those cases were assessed individually.

### Phylogenetic analysis of NAC TFs in *H*. *vulgare*, *O*. *sativa*, *B*. *distachyon and A*. *thaliana*

NAC domain (Pfam ID: NAM; PF02365) peptide sequences from barley (167), rice (139), *B*. *distachyon* (136) and *A*. *thaliana* NAC TFs (116) were aligned using MAFFT ver.7 with iterative refinement (G-INS-i) with“leave gappy regions” set to “Unalignlevel 0.8” [36,37]. A phylogenetic tree was constructed using the maximum likelihood method with 100 bootstrap repeats in MEGA 7.0 [38].

Further, the NAC domain peptide sequences from barley, rice, *B*. *distachyon* and *A*. *thaliana* were used for phylogenetic tree construction together with those of the Triticeae species common wheat (*Triticum aestivum*), *Aegilops tauschii*, wild emmer (*Triticum turgidum*), wild einkorn (*Triticum urartu*) and rye (*Secale cereale*), the Panicoideae species *Sorghum bicolor*, foxtail millet (*Setaria italica*), switchgrass (*Panicum virgatum*) and maize (*Zea mays*); the Chloridoideae species *Zoysia pacifica* the Bromelioideae species pineapple (*Ananas comosus*); the Zingiberales species banana (*Musa acuminate*); and the Commelinids species oil palm (*Elaeis guineensis*) (compare Table 1). The sequences were acquired from the Plant Transcription Factor Database v4.0 ([1,39]; http://planttfdb.cbi.pku.edu.cn/) for *A*. *tauschii*, *S*. *bicolor*, foxtail millet, switchgrass, maize, *Z*. *pacifica*, pineapple, banana and oil palm. For wild emmer, wild einkorn, wheat and rye, the protein sequences were obtained from the respective genome databases [40–42]. NAC domains were identified by HMMER search with the same setting as described above. An alignment of the NAC domains was carried out using MAFFT ver.7 with the “L-INS-i” algorithm. Due to the large size of the alignment, an approximately-maximum-likelihood phylogenetic tree was built from the alignment with FastTree version 2.1.10 run with options “-pseudo -spr 4 -mlacc 2 -slownni” for increased accuracy [43,44].

**Table 1:** Overview of the taxonomy and the number of NAC TFs in the species used in this study.

Species	Superorder	Order	Family	Subfamily	Tribe	Ploidy	Genome designation in allopolyploids	Total NAC TFs	NAC-d	NAC d-9	Grain NACs	References
*Arabidopsis thaliana*	*Eudicotyledons*	*Brassicales*	*Brassicaceae*	*Brassicaceae*	*Camelineae*	2		117	14	2	0	[1,28–30,39]
*Elaeis guineensis (Oil palm)*	*Monocotyledons (Liliopsida)*	*Arecales*	*Arecaceae*	* Arecoideae*	*Cocoseae*	2		170	34	5	0	[1,39]
*Musa acuminata (Banana)*	*Monocotyledons (Liliopsida)*	*Zingiberales*	*Musaceae*	* *	* *	2		168	41	10	0	[1,39]
*Ananas comosus (Pineapple)*	*Monocotyledons (Liliopsida)*	Poales	*Bromeliaceae*	*Bromelioideae*	* *	2		73	15	4	0	[1,39]
*Zoysia pacifica*	*Monocotyledons (Liliopsida)*	Poales	*Poaceae*	*Chloridoideae*	*Zoysieae*	4	All	200	49	16	5	[1,39]
							A (Odd)	89	19	6	3	
							B (Even)	94	26	7	1	
							U	17	4	3	1	
*Panicum virgatum (switchgrass)*	*Monocotyledons (Liliopsida)*	Poales	*Poaceae*	*Panicoideae*	*Paniceae*	4	All	297	50	13	7	[1,39]
							K (a)	132	23	6	3	
							N (b)	146	25	7	4	
							U	19	2	0	0	
*Setaria italica (Foxtail millet)*	*Monocotyledons (Liliopsida)*	Poales	*Poaceae*	*Panicoideae*	*Paniceae*	2		134	23	7	3	[1,39]
*Sorghum bicolor*	*Monocotyledons (Liliopsida)*	Poales	*Poaceae*	*Panicoideae*	*Andropogoneae*	2		127	24	8	4	[1,39]
*Zea Mays (Mays)*	*Monocotyledons (Liliopsida)*	Poales	*Poaceae*	*Panicoideae*	*Andropogoneae*	2		131	24	7	2	[1,39]
*Oryza sativa (Rice)*	*Monocotyledons (Liliopsida)*	Poales	*Poaceae*	*Oryzoideae*	*Oryzeae*	2		151	27	8	2	[1,9,27,28,32,39]
*Brachypodium distachyon*	*Monocotyledons (Liliopsida)*	Poales	*Poaceae*	*Pooideae*	*Brachypodieae*	2		136	20	7	4	[1,6,31,32,39]
*Hordeum vulgare (Barley)*	*Monocotyledons (Liliopsida)*	Poales	*Poaceae*	*Pooideae*	*Triticeae*	2		167	29	16	6	This study, [1,35,39,45,46]
*Secale cereale (Rye)*	*Monocotyledons (Liliopsida)*	Poales	*Poaceae*	*Pooideae*	*Triticeae*	2		109	26	16	9	[1,39]
*Triticum aestivum (Bread wheat)*	*Monocotyledons (Liliopsida)*	Poales	*Poaceae*	*Pooideae*	*Triticeae*	6	All	436	74	35	19	[1,39]
							A	145	24	12	6	
							B	134	23	10	6	
							D	142	22	11	6	
							U	15	5	2	1	
*Aegilops tauschii*	*Monocotyledons (Liliopsida)*	Poales	*Poaceae*	*Pooideae*	*Triticeae*	2		114	28	14		[1,39]
*Triticum turgidum (Wild emmer)*	*Monocotyledons (Liliopsida)*	Poales	*Poaceae*	*Pooideae*	*Triticeae*	4	All	229	54	28		[1,39]
							A	115	26	14		
							B	110	27	13		
							U	4	1	1		
*Triticum urartu (Wild einkorn)*	*Monocotyledons (Liliopsida)*	Poales	*Poaceae*	*Pooideae*	*Triticeae*	2		72	10	2		[1,39]

In this tree, the NAC-d-9 subfamily TFs were identified following the nomenclature introduced by [14] and subjected to a refined analysis. Only sequences containing at least subdomains A to D were considered. Wild einkorn, *A*. *tauschii* and wild emmer were not included in the refined analysis, as common wheat was represented. NAC domain peptide sequences were aligned using MAFFT ver.7 with iterative refinement (G-INS-i) set to “leave gappy regions” with “Unalignlevel 0.8” [36,37,47]. The phylogenetic tree was constructed using maximum likelihood with 100 bootstrap repeats in MEGA 7.0 [38].

### Gene expression, promoter region and C-terminal sequence analysis

Gene expression analysis for all the barley NAC genes across 15 tissues/developmental stages was based on gene expression data (FPKM values from RNAseq experiments) [22,35] obtained from the Barlex genome explorer, and heatmaps were constructed using the heatmap.2 function of the R package gplots [45,48]. Eleven genes with no expression across all samples were removed before heatmap construction. Values of other individual samples with expression value = 0 were changed to the minimum positive values of all samples before log transformation. Genes were either ordered in the heatmap according to their NAC subfamily (a to h), and within each subfamily ordered by descending total expression values across all samples, or they were ordered according to a hierarchical clustering of the log2 FPKM values, using a Pearson correlation distance function. The number of basic clusters was determined by the Nbclust R package [49]. An additional heatmap for wheat NAC-d-9 genes across 25 different samples/tissues types was also constructed, using RNA-seq data compiled by [50].

All the putative NAC promoter sequences were obtained from Ensembl Plants gene annotation ([33]; https://plants.ensembl.org, accessed 24 April 2018), confined to the 1 kb upstream region from ATG start codon of each gene. The cis-regulatory analysis of Grain-HvNAC promoters (HORVU4Hr1G089450, HORVU3Hr1G014090, HORVU7Hr1G039700, HORVU3Hr1G014100, HORVU7Hr1G031260, HORVU7Hr1G122680), senescence-associated HvNAC promoters (HORVU5Hr1G045640, HORVU2Hr1G017400, HORVU2Hr1G017380, HORVU5Hr1G074810, HORVU4Hr1G051360, HORVU2Hr1G080460, HORVU7Hr1G082420), and four house-keeping genes (HORVU7Hr1G074690, HORVU3Hr1G079700, HORVU1Hr1G081280, and HORVU1Hr1G002840) were determined using the PLACE database of motifs found in plant cis-acting regulatory DNA elements ([51]; https://sogo.dna.affrc.go.jp/cgi-bin/sogo.cgi?lang=en&pj=640&action=page&page=newplace). Also, the orthologous Grain-NAC promoters in wheat (TRIAECS42_7DS_TGACv1_623144_AA2049980, TRIAECS42_3DS_TGACv1_273115_AA0928510, TRIAECS42_7DS_TGACv1_623437_AA2053190, TRIAECS42_7DS_TGACv1_623146_AA2050060, TRIAECS42_3AS_TGACv1_210879_AA0680650, TRIAECS42_7DL_TGACv1_602807_AA1969110), maize (GRMZM2G154182, GRMZM2G062650) and rice (ONAC20; Os01g0104500 and ONAC26; Os01g0393100) were included. Only identical boxes were considered in the search of motif for the promoter sequences. EIN3 motifs were found in the PlantPAN 2.0 database ([52]; http://plantpan2.itps.ncku.edu.tw/promoter.php).

The encoded C-terminal protein sequences (defined to start at pos. 6 after the conserved cysteine residue of the NAC E-subdomain) of the barley, rice and maize Grain-NACs and senescence-associated HvNACs mentioned above were analyzed using the MEME software ([53]; http://meme-suite.org/, number of motifs=6, max. motif length=12, accessed April 24, 2018) for the search of conserved motifs. Alignment of NAC promoter and C-terminal sequences was done in CLC Main Workbench 7.9.1 using the “very accurate” algorithm (QIAGEN Aarhus, Denmark; https://www.qiagenbioinformatics.com/).

## Results and discussions

### Identification of HvNACs in the barley reference genome assembly

Recently, the NAC TF family of hexaploid wheat was thoroughly categorized [7], based on the recent update of the wheat genome assembly [21]. The latest study of the barley NAC TF gene family however dates back to 2011 and identified only 48 different NAC TF encoding genes [46]. Due to the limited sequence resources available at that time, this list was however bound to be incomplete. The recently published barley reference genome assembly [22] now enabled us to characterize the full set of NAC TF genes present in barley. In total, 167 barley NAC TFs (S1 Table) were identified. One hundred thirty sequences were found by keyword search; hence they were annotated as NAC TFs. Subsequent BLASTp analysis and a HMMER search based on the Hidden Markov model for the Pfam domain PF02365 characterizing the conserved N-terminus of the NAC TFs identified 11 and 2 additional HvNACs respectively in the peptide sequences representing annotated gene models. Furthermore, tBLASTn against the genomic sequence was done using the protein sequences of all HvNACs identified so far as query. This approach identified 24 additional genomic regions. While 11 of those were overlapping gene annotations, 13 genomic regions were not. One of the HvNACs, which was annotated in the previous genome assembly [35] and described as HvNAC026 by Christiansen et al. (2011) [46] was, however not identified by any of our searches in the new genome assembly [22].

**Fig 1:**
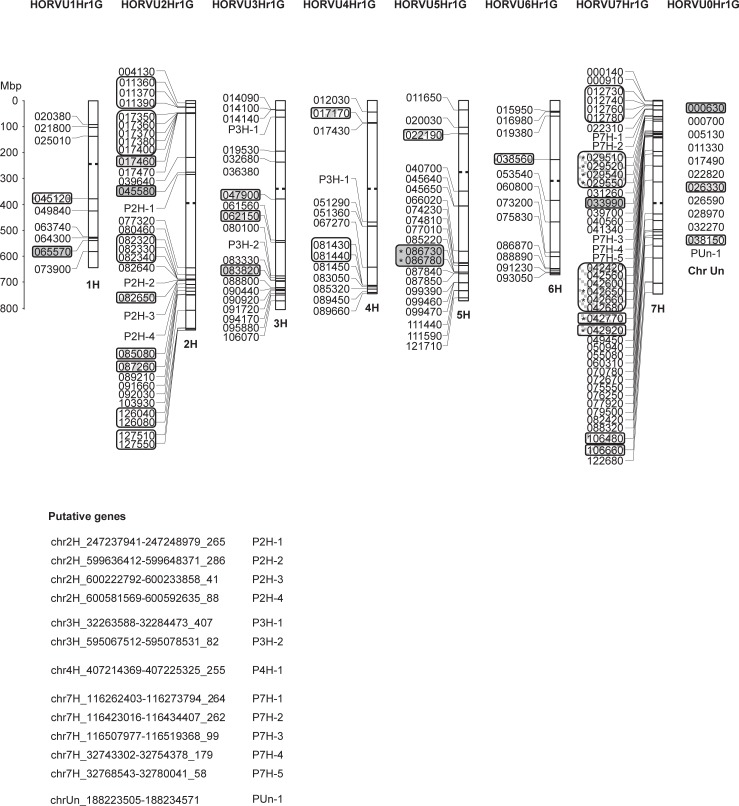
Chromosomal location of NAC TF genes in barley. The location of 154 barley NAC TF encoding genes and 13 genomic regions potentially encoding NAC TFs on the seven barley chromosomes are illustrated. The common prefixes of gene IDs (ex. HORVU1Hr1G) are shown on the top of each chromosome. Asterisks indicate that the NAC domains were not found within the published gene models but only in predicted ORFs in the gene regions. ChrUn refers to an unknown chromosomal location in the reference genome. Tandem duplications are framed while segmental duplications are marked with filled rectangles in matching shadings. Centromere positions are shown by dotted lines.

Fig 1 shows the distribution of NACs in the barley genome. More than 30 NAC TF genes were found on chromosomes 2H and 7H, while chromosome 1H carries the lowest number of only 9 genes. However, no specific pattern of distribution was observed. Among the 167 loci, 10 tandem duplications and 8 segmental duplications were identified (Fig 1). Most of the tandem duplications were found on chromosomes 2H and 7H, which also bear the highest number of NAC TF genes. Segmental duplications were found on all chromosomes.

The NAC TF encoding genes identified in this study tend to be located in the distal regions of the chromosomes, where also many of the duplication events were observed (Fig 1). This unequal localization of NAC TF genes across chromosomes is not surprising as the gene density in the distal regions of the chromosomes is increased [22]. In wheat, it has further been shown that - while single loci are more frequent in proximal parts of the chromosomes, gene duplications often occur in the distal regions [54].

### Barley NAC TFs can be classified into different subfamilies

Shen et al. (2009) [14] classified NAC TFs into eight subfamilies, designated as NAC-a to NAC-h, and suggested that these subfamilies might have distinct functions. For instance, many NAC-a subfamily members function in stress responses and hormone signalling [55–59], while the NAC-b classified factors are mainly involved in ER stress regulation and cell cycling [60]. The NAC-c subfamily contains NACs involved in secondary cell wall biosynthesis and PCD [14,61–63]. NAC-d TFs appear to have a role in organ initiation and differentiation like shoot apical meristem development or the formation of lateral roots and flowers [14,64–66]. Several NAC-d subfamily members are further involved in the senescing process both under normal and stress conditions [67–73].

**Fig 2:**
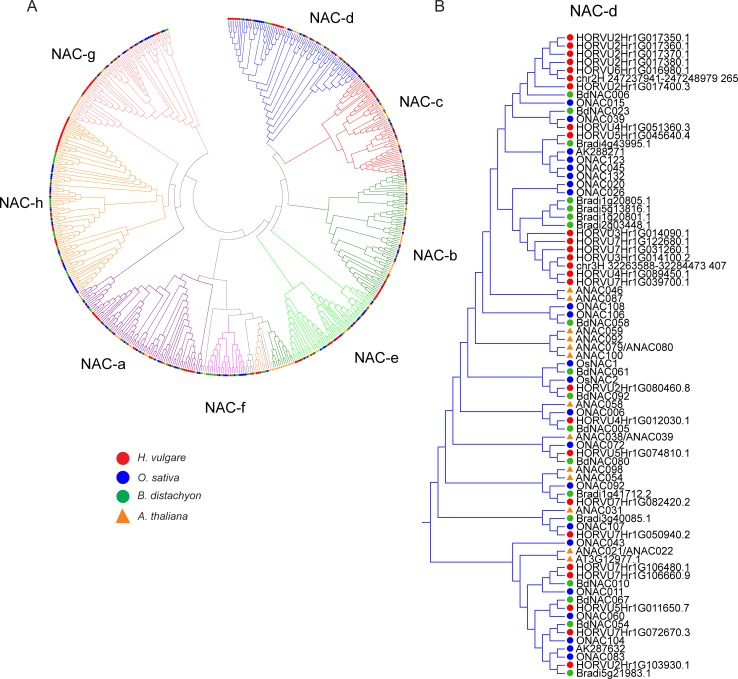
Phylogenetic analysis of the NAC TFs from *H*. *vulgare*, *O*. *sativa*, *B*. *distachyon* and *A*. *thaliana*. (A) Phylogenetic tree of the NAC domain peptide sequences from *H*. *vulgare*, *O*. *sativa*, *B*. *distachyon* and *A*. *thaliana*. The tree was constructed using the Maximum Likelihood method with 100 bootstrap repetitions. The subfamilies NAC-a to NAC-h were assigned using the nomenclature introduced by Shen et al. (2009) [14]. Each species is represented by symbols with different colours. (B) Subtree of the NAC-d subfamily. The subtrees of the other subfamilies are shown in S2 Fig.

In order to classify the newly identified barley NAC TFs according to the nomenclature introduced by Shen et al. (2009) [14], the peptide sequences spanning the NAC domains of the 167 HvNACs identified were used together with the NAC domains from *O*. *sativa*, *A*. *thaliana* and *B*. *distachyon* to construct a phylogenetic tree (Fig 2; S2 Fig; S2 Table). Sequences from barley, rice and *B*. *distachyon* were found to group into clusters of orthologues, while *A*. *thaliana* NAC TFs often formed their own subclades reflecting the larger distance between monocot and dicot NAC domains. The TF subfamilies corresponding to NAC-c, NAC-d and NAC-f could be identified unequivocally, meaning that all sequences clustered into the expected subfamilies following Shen et al. [14]. Minor discrepancies were, however, found within the other subfamilies. Notably, five members of the NAC-b subfamily were found to possess transmembrane domains, an observation that has been described before [60,74]. In the current study, sequences failed to cluster unambiguously into subfamilies NAC-f and NAC-g [14], as some sequences previously shown to belong into these subfamilies [14], were found elsewhere in the current study (Fig 2), which might be due to differences in the sequences used as well as in alignment and tree construction settings.

### Expression patterns of barley NAC TF genes

**Fig 3:**
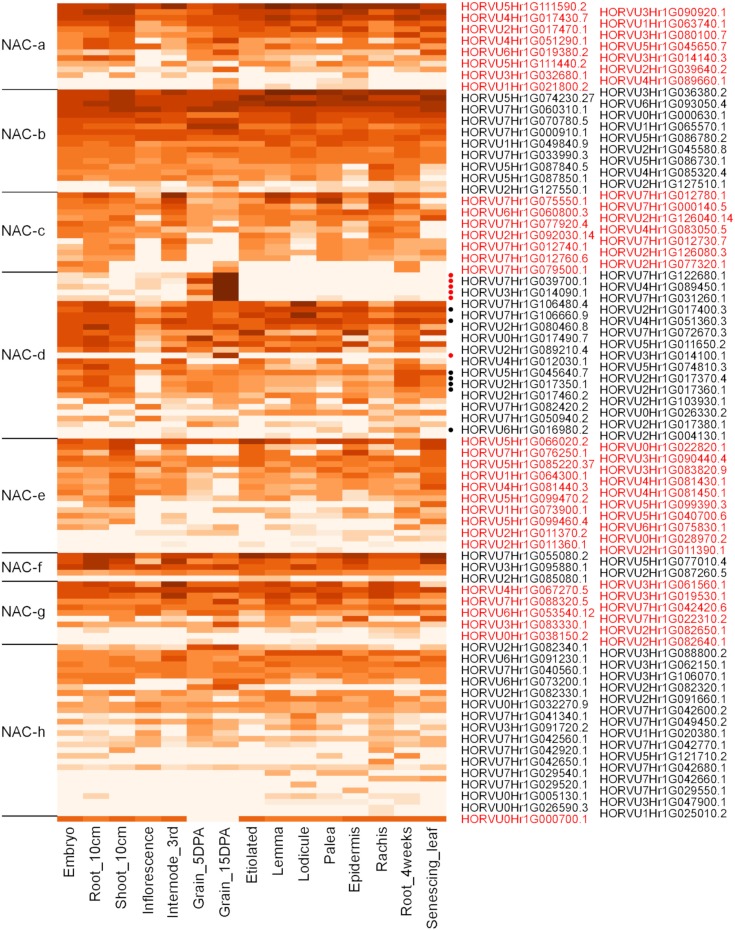
Heatmap of gene expression values (log2 FPMK values) for HvNAC genes across 15 samples representing different tissues and developmental stages. The RNA-seq expression data are taken from Mascher et al. (2017) [22], omitting one sample from the inflorescence (referred to as INF1) as this sample was characterized by very low expression levels. The NAC genes are ordered according to their respective subfamily classification (designated as a to h [14]), and within each subfamily, genes are ordered by descending total expression values across all samples. The last gene, HORVU0Hr1G000700.1, could not be associated to any of the subfamilies. Alternating colouring of gene names delineates subfamilies. On the right, filled circles mark the NAC-d-9 subgroup, and red filled circles further mark the six Grain-NAC genes within the NAC-d-9 subgroup.

Apart from phylogenetic relationships, gene expression patterns can hint at the function of specific genes as well. Therefore, a heatmap of expression levels for all barley NAC TF genes, for which gene models can be found in the barley reference assembly, was constructed. The heatmap was based on publicly available RNAseq data from 15 samples representing different tissues and developmental stages [22,35] and is shown in Fig 3. As also observed in wheat [7], we overall found similar expression patterns within the different subfamilies. The NAC-d subfamily however depicts a striking exception, as its members clearly have two different patterns of expression. While most genes in the NAC-d-9 subfamily are expressed over a wide range of different samples, six genes (HORVU4Hr1G089450.1, HORVU7Hr1G039700.1, HORVU7Hr1G122680.1, HORVU3Hr1G014090.1, HORVU7Hr1G031260.1, HORVU3Hr1G014100.1) show a very strong and almost exclusive expression in developing grains (Fig 3).

Notably, five of these six genes depict the five most highly expressed genes across all samples. This observation prompted us to explore the group of highly expressed grain-specific NAC-d genes further. A heatmap based on hierarchical clustering of expression levels across the 15 different samples was constructed at the overall expression level to detect NAC genes from other subfamilies with grain-specific expression (S1 Fig). This effort divided the NAC gene expression patterns into 14 clusters. The NAC-d genes with grain specific expression clustered in clade #14, which consists of genes that have their highest expression levels in the developing caryopsis. Indeed, there was one additional gene, HORVU2Hr1G082320.2, that showed close to exclusive expression in the caryopsis, but at a much lower level in both of the two caryopsis samples included in the analysis than the NAC-d genes with grain specific expression. Phylogenetically, this gene falls into the NAC-h subfamily (Fig 2; S2 Fig; S2 Table), the members of which are characterized by diverging, weaker NAC domains [14]. The other genes in this cluster however did not show an exclusive expression in the grain. Hence, the six NAC-d genes referred to above stood out as an exceptional set of genes with very high and grain-specific expression, making a detailed analysis of the NAC-d subfamily relevant.

### Evolution of the NAC-d subfamily in monocots

**Fig 4:**
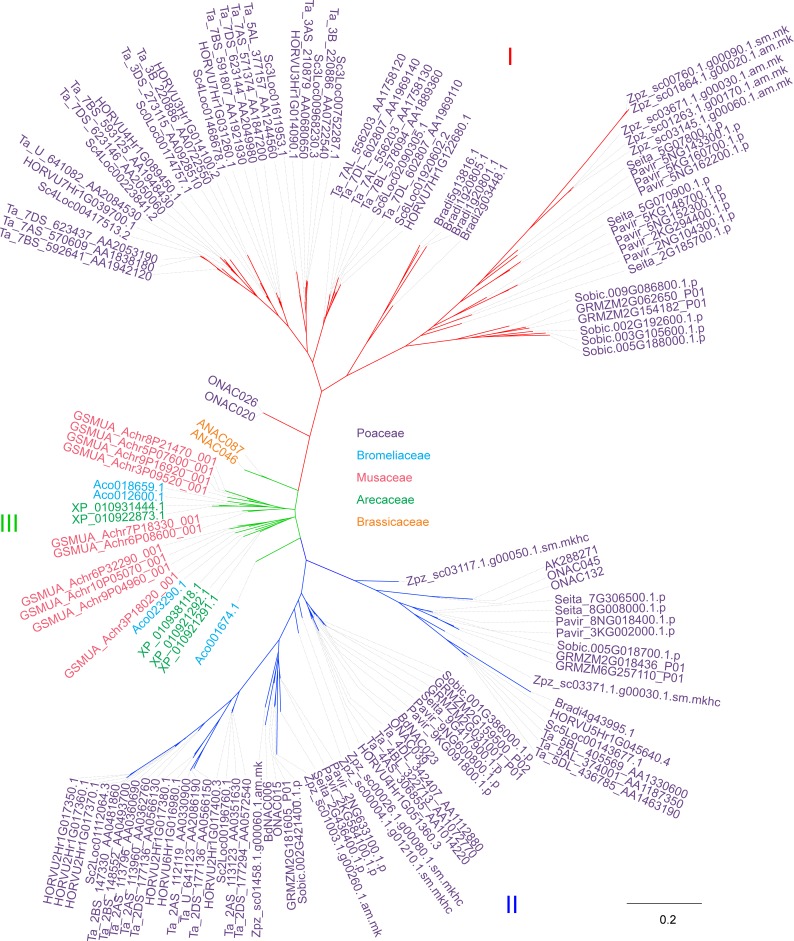
Phylogenetic analysis of the NAC-d-9 subgroup in monocotyledonous species and *Arabidopsis thaliana*. Phylogenetic analysis of the NAC-d-9 subgroup in barley (*H*. *vulgare*), common wheat (*T*. *aestivum*), rye (*S*. *cereale*), rice (*O*. *sativa*), *B*. *distachyon*, *S*. *bicolor*, foxtail millet (*S*. *italica*), Switchgrass (*P*. *virgatum*), maize (*Z*. *mays*), *Z*. *pacifica*, pineapple (*A*. *comosus*), banana (*M*. *acuminata*), oil palm (*E*. *guineensis*) and *A*. *thaliana* using the respective NAC domain peptide sequences. The tree was constructed using the NAC domain protein sequences by the Maximum Likelihood method with 100 bootstrap repetitions. Three subclades, designated as I, II and III, were identified and are shown in different colours. The scale bar shows evolutional distance of 0.2 aa substitution in the sequences.

Next, a comprehensive analysis of the evolution of the NAC-d subfamily in monocots was performed. To this end, a phylogenetic tree using the NAC domain sequences from 17 monocot species, for which fully sequenced genomes are available, and *A*. *thaliana* was constructed (S3 Fig). The monocotyledonous species included in this analysis were barley (*H*. *vulgare*), common wheat (*T*. *aestivum*), *A*. *tauschii*, wild emmer (*T*. *turgidum*), wild einkorn (*T*. *urartu*), rye (*S*. *cereale*), rice (*O*. *sativa*), *B*. *distachyon*, *S*. *bicolor*, foxtail millet (*S*. *italica*), switchgrass (*P*. *virgatum*), maize (*Z*. *mays*), *Z*. *pacifica*, pineapple (*A*. *comosus*), banana (*M*. *acuminata*) and oil palm (*E*. *guineensis*). The systematic classification of the species can be found in Table 1. The phylogenetic analysis enabled the identification of the NAC-d subfamily (S3 Fig) in all species mentioned and it became apparent that the NAC-d-9 subgroup [14] has expanded during the evolution of monocots (Fig 4). In particular, the Triticeae species were found to have nearly twice as many NAC TFs in this subgroup as the other monocot species.

For further analysis, a phylogenetic tree based on the peptide sequences of the NAC domains of the d-9 NAC TFs was constructed. *A*. *tauschii*, wild emmer and wild einkorn were omitted from the analysis, as common hexaploid wheat was included. The tree branched into three clades designated as I, II and III (Fig 4; S3 Table). Clade I contains the six barley NAC-d-9 genes with almost exclusive expression in the grain. Both clade I and II were found to be specific to Poaceae, while clade III contained NAC TFs from monocotyledonous species not belonging to the Poaceae family and from *A*. *thaliana*. Notably, several of the NAC-TFs in clade II and III are expressed and up-regulated in senescing leaves [16,69,73,75] and are further referred to as senescence associated NAC TFs in this study. The observation of two Poaceae specific subclades within NAC-d-9 hints to a diversification of this specific subgroup after the separation of the Poaceae family from other families in the order of Poales. Furthermore, an increased number of NAC TFs in the d-9 subgroup of the Triticeae implies an expansion of NAC TFs in this specific subgroup during the evolution of Triticeae (Table 1).

### A subclade of NAC-d-9 TFs shows grain specific expression

Notably, the six barley d-9 NACs with almost exclusive expression in the developing grain fell into the same clade (Figs 3 and 4; S3 Table) as a number of genes from other Poaceae species for which high expression in the grain has been observed previously; however, without making clear associations with a Poaceae-specific phylogenetic clade as we describe it here (e.g. [46,76–78]). The wheat homologs in this clade for instance showed a strong and exclusive expression patterns in wheat grain tissues (S4 Fig; [7,21,50]. The two rice NAC TFs, ONAC020 and ONAC026, that fell into clade I of the NAC-d-9 subgroup as well, are known to be highly expressed specifically in grains during maturation. Notably, they are associated with grain size and weight phenotypes [79]. Further, two maize NACs, which also fell into clade I, have previously been characterized as grain specific: NRP1 (GRMZM2G062650) and ZmNAC4 (GRMZM2G154182) [65,80]. We hence show here that a group of NAC TFs, that are highly and specifically expressed in grains, forms a distinct clade within the NAC-d-9 subgroup. We therefore propose to designate these TFs as Grain-NACs. Grain-NACs are specific for the Poaceae family of monocotyledonous plants, as they were found to be absent from the Bromeliaceae species *A*. *comosus* and from the Arecales and Zingiberales species included in this study as well as from the dicotyledonous *A*. *thaliana*.

Due to their organ specific expression and as NAC-d TFs are often involved in organ formation and differentiation, Grain-NACs possibly have a role in the development of the caryopsis, the fruit typical for the Poaceae family of grasses, and more specifically its storage tissue, the starchy endosperm. Notably, it is particularly the endosperm that depicts the value of such important cereal crops as wheat, rice, maize, or barley for human nutrition and for countless industrial applications. Its main function is to provide nutrients to the developing and later germinating embryo. The Poaceae endosperm is, in contrast to many species, including *A*. *thaliana*, a persistent seed structure. Endosperm development includes several distinct phases: Upon double fertilization, syncytium formation and subsequently cellularization occurs. This is followed by cell differentiation and the periods of mitosis, endoreplication and storage compound accumulation and finally maturation, including PCD, dormancy and desiccation (summarized in [81]).

Notably, several reports on gene expression in developing grains have included Grain-NAC genes, still without characterizing them as a Poaceae-specific clade of NAC genes. Retrospectively, this data can be used to relate the Grain-NAC gene expression profiles to the specific stages of endosperm development. Thus, expression of the Grain-NAC genes appears to be initiated 7-10 days after flowering in both barley and wheat [76–78] just before the start of storage compound accumulation [82]. Hence, there is high expression of the Grain-NAC genes throughout most of the grain filling period, paralleling the accumulation of starch and storage proteins. Maximal expression of Grain-NACs in barley and wheat occurred 15-25 days after flowering. Similar patterns were observed for the two maize Grain-NAC genes [65,80]. Notably, in maize and wheat the earliest occurrence of PCD in the endosperm is around 16 DAP [83–85]. It has been suggested that ethylene is involved in PCD during endosperm development [86] and NACs are known to play a role in ethylene signalling [87–89].

### Cis-regulatory elements leading to grain-specific expression in Grain-NACs

**Fig 5:**
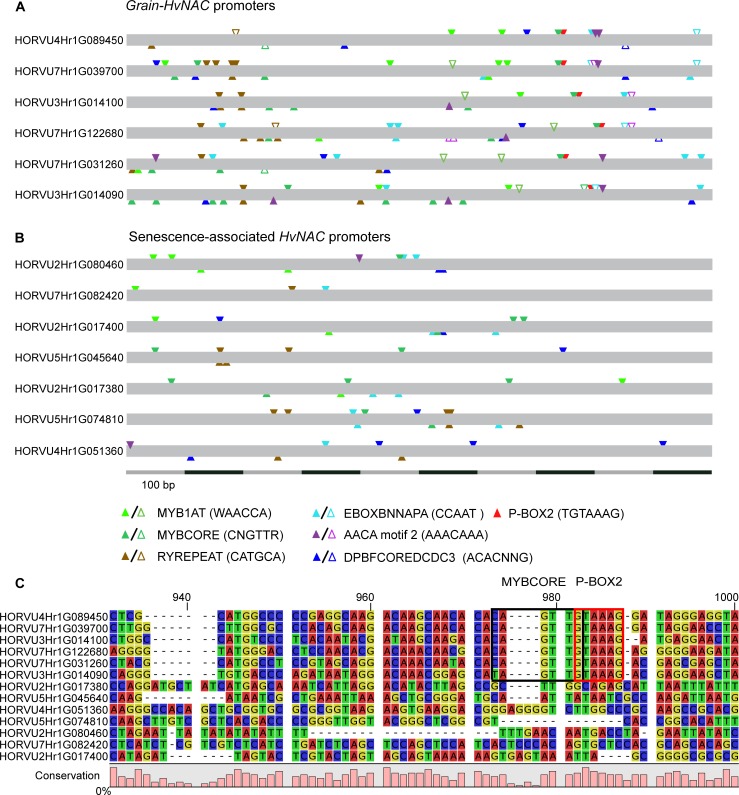
Cis-regulatory element annotation and alignment of promoter sequences from Grain-HvNAC and senescence-associated HvNAC genes. The positions of seven conserved cis-elements putatively involved in seed development are shown in six Grain-HvNAC promoters (A) and seven senescence-associated HvNAC promoters (B). Identical seed-specific motifs are represented by filled triangles, while similar motifs (elements with a SNP variation) are represented by open triangles. Triangles on the upper and lower side represent the orientation on positive and negative strands, respectively. A partial alignment of Grain-HvNAC and senescence-associated HvNAC promoters is shown in (C). A black and red box indicate a MYBCORE and P-BOX2 motif, respectively, which together form an eleven nucleotide long motif conserved across the Grain-HvNAC promoters.

As Grain-NAC genes are exclusively and strongly expressed during seed development the question about which cis-regulatory elements in promoter regions are involved in grain specificity is obvious. To explore this, we compared the occurrence of specific promoter motifs involved in seed development both in Grain-NAC and senescence-associated NAC promoter sequences. Although these are phylogenetically closely related, their expression patterns differ considerably (Fig 3). Seventeen distinct cis-regulatory elements involved in seed development were found (S4 Table). Amongst them, P-BOX2, RYREPEAT, EBOXBNAPA, DPBFCORE, AACA motif 2, MYB1AT and MYBCORE elements were conserved and exhibited similar patterns across all six Grain-HvNAC promoters (Fig 5A). In total, 197 motifs were found in the six Grain-HvNAC promoters, an average of about 33 motifs per sequence, while only 138 of such motifs were found in the seven senescence-associated HvNAC promoters, an average of about 20 motifs per sequence (S4 Table). A statistical test showed that promoter motifs associated with seed development were significantly over-represented in Grain-HvNAC promoter sequences compared with senescence-associated HvNAC promoters and also with a group of four unrelated house-keeping genes (Fig 5A and 5B, S4 Table).

The P-BOX2 element or prolamin box was found to be exclusively present in Grain-HvNAC promoters while absent from senescence-associated HvNAC promoters (Fig 5A and 5B). The P-BOX2 element occurred only once per Grain-HvNAC promoter. This box is essential for regulating the expression of seed storage proteins (SSPs) both in barley and wheat [90]. Further, an alignment of Grain-HvNAC and senescence-associated HvNAC promoter sequences identified a fully conserved motif of eleven nucleotides (Fig 5C), representing overlapping P-BOX2 and MYBCORE motifs, in the Grain-HvNAC promoters, which is absent in the promoters of the senescence-associated HvNAC genes. The MYBCORE, as well as the AACA motif 2 and MYB1AT elements are specific binding sites for the R2R3-MYB TFs [91,92]. Among these, GAMYB, MCB1 and MYBS3 have an important role in activating endosperm specific genes during seed development [93–96]. The conserved eleven nucleotide core may hence be essential for the regulation of the grain specific expression of the Grain-NAC genes in barley. Only 54 nucleotides further downstream of this region another conserved region was found, corresponding to the EBOXBNAPA, AACA motif 2 and DPBFCOREDCDC3 (S5 Fig). EBOXBNAPA, conserved in SSP promoters, is critical for directing seed specific expression in *Brassica napus* [97]. DPBFCOREDCDC3 is an embryo specific element that interact with bZIP-TFs [86]. Finally, the AACA motif 2 is required for the high level of glutenin expression in the starchy endosperm [98]. Ravel et al. (2014) [99] observed a common regulatory framework of cis-elements in high molecular weight glutenin subunits (HMW-GS) gene promoters that regulate the transcription of SSP genes and hence partially explain their high expression level in several wheat varieties. Potentially the P-BOX2, RYREPEAT, EBOXBNAPA, AACA motif 2 and MYB1AT motifs depict a similar framework for Grain-HvNAC promoters, resulting in the high grain-specific expression levels shown in Fig 3. Notably, the same cis-elements described above for barley were further found with similar numbers in wheat, rice and maize Grain-NAC promoters (S6–S8 Figs, S5 Table). As discussed above, grain-specific expression of wheat and maize Grain-NAC genes has been observed, and Mathew et al. (2016) [79] showed that OsNAC20 and OsNAC26 are expressed specifically during rice seed development at extremely high levels.

Yet another motif conserved across the Grain-HvNAC promoters was the RYREPEAT motif (S5 Fig). This motif was found to be positioned distantly from the transcription initiation site when compared to the conserved motifs described above and it was present in multiple copies in some of the Grain-HvNAC promoters (Fig 5A). This motif is known to participate in the transcriptional activation of the endosperm specific genes *Hor2* and *Itr1* through binding of the FUS3 protein to RYREPEAT element in barley [100].

Notably, the RYREPEAT motif (CATGCA) is very similar to the EIN3 motif (ATGCAT). These DNA binding domains are often found together in the same promoter region. The EIN3 motif, as its name indicates, serves as a binding site for EIN3 and EIN3-like transcription factors [101,102], which are positive regulators in ethylene signaling [103]. Ethylene is a key regulator of plant senescence [104] and is also a mediator of PCD in the cereal endosperm [85]. The promoters of both the Grain-HvNAC and senescence-associated HvNAC genes characterized in this study display a high number of RYREPEAT/EIN3 motifs, however, with a higher number in the Grain-HvNAC genes (S4 Table). It hence appears plausible that the multiple copies of RYREPEAT/EIN3 motifs add to the differential expression levels between Grain-NAC and senescence-associated NAC genes.

Overall, our analysis of the Grain-HvNAC promoters showed that those likely are unrelated to promoters of the phylogenetically most closely related senescence-associated HvNAC genes (based on the NAC A-E subdomains). There is no apparent conservation of promoter motifs between these two groups (Fig 5), and upon alignment they did not show higher similarities to each other than to NAC promoters from other HvNAC subfamilies (data not shown). Hence, we conclude that the origin of the Grain-HvNAC promoters is different from that of the senescence-associated HvNAC genes, which apparently is associated with the evolutionary acquisition of seed specific motifs in the Grain-HvNAC promoters.

### DLN and NARD motifs are conserved in Grain-NACs as well as in senescence-associated NACs whereas conserved motifs in C-terminal parts differ considerably

The two rice NAC TFs, ONAC020 and ONAC026, that fell into the Grain-NAC subclade have been shown to comprise a DLN repressor motif in the subdomain B of their NAC domain, which is an EAR (Ethylene-responsive element binding factor-associated Amphiphilic Repression) motif functioning as repressors in various signalling pathway including ethylene [79,105,106], and a NAC repression domain (NARD) in their subdomain D [79,105,107]. Transactivation and transrepression assays in yeast demonstrated that these motifs act as repressors of the transactivation function [79,107]. In this study, we found that among the 141 NAC-d-9 sequences included in the analysis (Fig 4; S3 Table), 102 (72%) had a DLN motif and 135 (96%) a LVFY motif, which is the most conserved motif in the NARD domain (S6 Table). Notably, all NACs with a DLN motif also comprised a LVFY motif. This is contrasted by the fact that a NARD motif was found only in about half and a DLN motif only in one fifth of the total population of NACs when all the subfamilies were included. The observation that more than half of all NAC-d-9 TFs comprise DLN motifs hints to a function involved in ethylene signalling and regulation. Furthermore, it has been suggested that the overall activity of NAC TFs is determined through interaction between the NARD repressor domain and an activation domain at C-terminus [107]. Since the NARD domain is present in most of the members of the NAC-d-9 subgroup, these might share similar regulative mechanisms.

**Fig 6:**
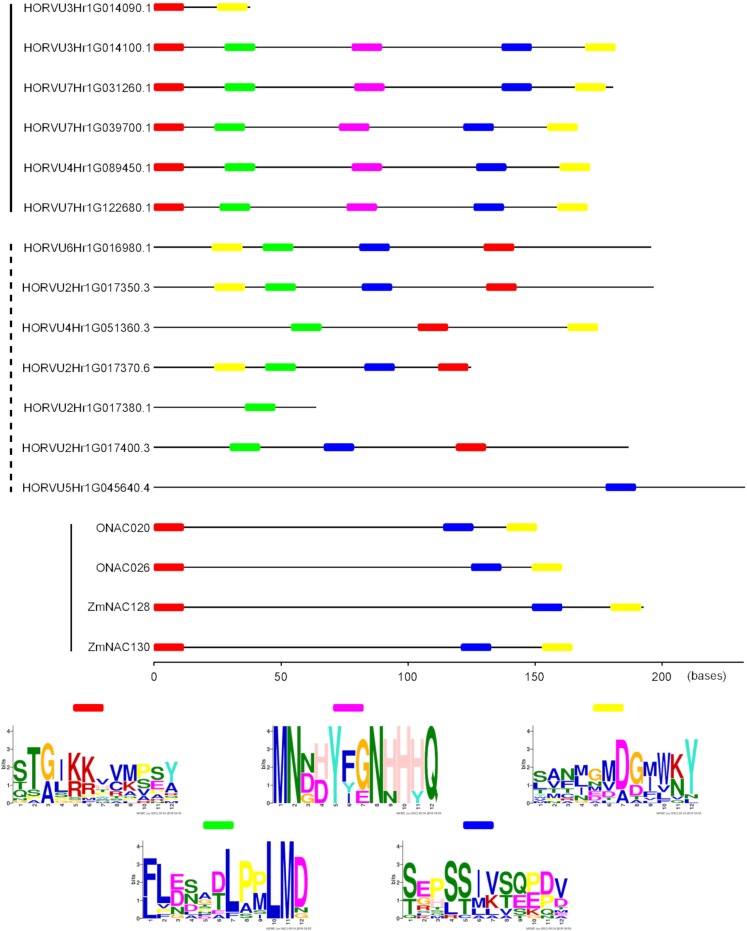
Positions of conserved short motifs in the C-terminal parts of NAC-d-9 protein sequences, for 13 barley, 2 rice, and 2 maize proteins. The C-terminal part was defined to start at position 6 after the conserved cysteine residue of the E subdomain. Conserved motifs were identified by the MEME software ([53]; http://www.meme-suite.org) with the settings: number of motifs – 6, max. length of motif – 12. WebLogos of the identified motifs are shown. On the left, full lines indicate Grain-NAC sequences, and the dotted line indicates senescence-associated HvNAC sequences. Rice Grain-NAC TFs: ONAC20 (LOC_Os01g01470.1), ONAC26 (LOC_Os01g29840.1). Maize Grain-NAC TFs: ZmNAC128 (GRMZM2G062650), ZmNAC130 (GRMZM2G154182).

In contrast to the similarities observed in the N-terminus, the C-terminal parts of the NAC-d-9 members differ considerably: Apart from HORVU3Hr1G014090, which has a short, apparently truncated C-terminus of only 38 aa, the encoded Grain-HvNAC protein sequences have relatively conserved C-terminal parts, around 175 aa long, with 51.5-64.5% aa identity among each other. When comparing the five long Grain-HvNACs with the senescence-associated NAC-d-9 members, the intergroup amino acid identity range was 13.6-25.0% for the C-terminal parts, whereas this range for the NAC domain (A-E subdomains) was 51.3-66.8%. For both regions of the sequence, the Grain-HvNACs showed the highest level of similarity with the senescence-associated gene HORVU4Hr1G051360 (HvNAC013 in [73]). The intragroup conservation of the Grain-HvNAC C-termini probably reflects relatively recent duplication events giving rise to the expanded Triticeae clade of Grain-NACs. It was shown previously that the intrinsically disordered C-termini of NAC TFs contain short conserved motifs of possible importance for the interaction characteristics of the TFs [108]. Accordingly, an analysis for conserved motifs across the five long Grain-HvNAC C-terminal sequences using the MEME software ([53]; number of motifs=6, max. motif length=12) showed high conservation of at least five short motifs (Fig 6), and three of these motifs (a proximal and two distal) were conserved across to the rice and maize Grain-NACs. When including the senescence-associated HvNACs of the NAC-d-9 subgroup, only one motif showed conservation across the two subclades with respect to both sequence and relative position. Hence, in contrast to the N-terminal NAC domain, the C-termini are rather diversified, which is a typical characteristic of NAC TFs [14]. Albeit diverse, the C-terminal part appears to function as a transcription regulatory domain and, hence, it is important for fine-tuning of the transcriptional activity of the NAC TFs [30].

### Possible roles of the Grain-NAC TFs

**Fig 7:**
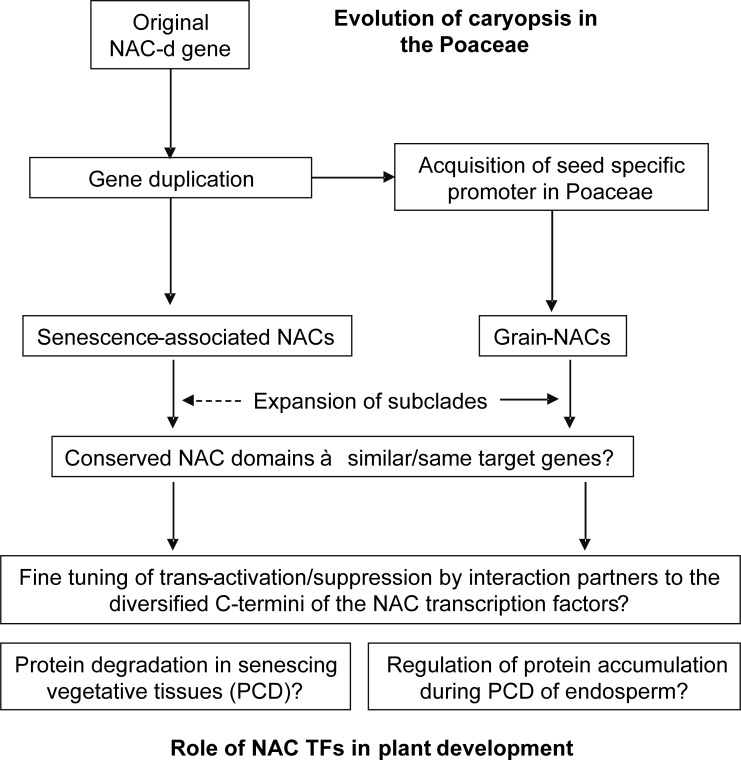
Model for the evolution of the Grain-NAC genes within the NAC-d-9 subgroup and their potential roles in grain/endosperm development. The model describes the possible events leading to the putative neo-functionalization of the Grain-NAC TFs relative to the senescence-associated NAC-d-9 members during evolution of the caryopsis of the Poaceae.

From our analysis of the Grain-NAC TFs of the Poaceae, and particularly the Triticeae, an interesting picture arises with respect to evolution of the Poaceae and the processes involved in grain filling and maturation of the caryopsis. A model of this is presented in Fig 7. First, the divergence of the Grain-NACs within the NAC-d-9 subclade of NAC genes appears to have taken place soon after, or during, the formation of the Poaceae, since they occur in all Poaceae species included in the analysis, but not outside the Poaceae. Our analysis of Grain-NAC promoter sequences revealed that an event must have taken place in which a copy of a NAC-d-9 coding sequence was combined with a promoter sequence mediating strong seed/endosperm specific expression. We hypothesize that this event could have contributed specifically to the evolution of the caryopsis, typically characterized by a starchy and dry endosperm at maturity [81]. Furthermore, we observed an expansion of the Grain-NAC clade within the Triticeae compared with other Poaceae species, e.g. rice and maize. A major radiation of Triticeae species (including barley and wheat) appears to have taken place 6.1-9.2 MYA in the Mediterranean area under a climate with cool winters and dry summers [109]. The action of the highly expressed Grain-NAC genes, enhanced by the gene duplications, might have been important for proper, i.e. fast, maturation in these dry environments, similar to contemporary Mediterranean environments.

Second, our analysis shows that there is a close phylogenetic relationship between the Grain-NACs and senescence-associated NACs of the NAC-d subfamily, based on a high degree of similarity among their respective conserved NAC domains. Since the NAC domains confer the DNA binding specificity [12], this similarity indicates that the Grain-NAC TFs and the senescence-associated NACd TFs also target similar down-stream genes. The identity of these genes is not firmly established, but there are many indications (see [16]) that they belong to the battery of genes encoding degradation factors involved in the senescence processes, e.g. proteases. The senescence process depicts a PCD event, in which a controlled degradation of contents in affected cells is completed [18]. Notably, the grain filling and maturation process in the cereal caryopsis is also characterized by a controlled PCD process [85]. Hence, we see a parallel between the leaf senescence process and the grain maturation process in the strong upregulation of NAC TFs accompanying PCD. The latter takes place in the endosperm towards the end of development while the machinery for starch and storage protein biosynthesis is still running to fill up the endosperm with storage compounds. Ethylene appears to be a strong regulator of the endosperm PCD [85], and the occurrence of binding signatures for EIN3, a central factor in ethylene signalling [110], within the many RYREPEAT motifs of Grain-HvNAC promoters (Fig 5A) might indicate an involvement of the Grain-NAC TFs in the ethylene regulation of endosperm PCD. Interestingly, EIN3 is also suggested to be a central up-stream regulator of senescence-associated NAC TFs in leaves [111], supported by the occurrence of a considerable number of RYREPEAT elements also in promoters of the senescence-associated NAC-d-9 genes, although at a lower number than for the Grain-NAC genes.

Further experiments are required in order to firmly establish the down-stream target genes of the Grain-NAC TFs. Even though the Grain-NAC and senescence-associated NAC-d TFs presumably recognize similar target promoter sequences, the actual outcome in terms of trans-activated genes might be considerably influenced by transcriptional interaction partners, possibly involving the interaction motifs of the diversified C-terminal parts of the NAC TFs [112]. Furthermore, the occurrence of the repressive DLN and NARD motifs in the NAC domain probably adds to the complexity of the regulatory role of Grain-NAC TFs during grain development. Hence, the degree of neo-functionalization of the Grain-NAC TFs compared with the senescence-associated NAC-d-9 members remains to be firmly established.

## Conclusions

The update of the barley NAC TF family performed in this work resulted in a total number of 167 members, i.e. in a range similar to that of other cereals such as rice. Most of the members could be allocated to the eight subfamilies a to h, as defined by Shen et al. (2009) [14]. This also reflects the quality of the new assembly of the barley genome [22], even though a number of the gene models still showed partial or truncated NAC TFs. Starting out from gene expression profiles, our analysis directed us towards a subclade of genes in the NAC-d subfamily deviating strongly from other NAC-d gene with respect to their expression patterns, as they showed strong and exclusive expression in the developing caryopsis coinciding with grain filling and the occurrence of PCD in the endosperm. We propose to designate this subclade of NAC-d TFs as Grain-NACs. Promoter analysis of the Grain-NAC genes revealed the occurrence of a number of cis-elements that are known to drive seed-specific expression of genes. Furthermore, phylogenetic analyses showed that the Grain-NAC subclade is specific for the Poaceae species. Based on this, we propose that the encoded Grain-NAC TFs might have played a role in the evolution of the caryopsis, i.e. the specialized fruit of the Poaceae with its typical dry and starchy endosperm.

## Supporting information

S1 Fig Heatmap of gene expression values (log2 FPMK values) for HvNAC genes across 15 samples representing different tissues and developmental stages. The RNA-seq expression data are taken from Mascher et al. (2017) [22], omitting one sample from the inflorescence (referred to as INF1) as this sample was characterized by very low expression levels. Grain-NACs are marked in red. A Pearson correlation distance function was used in the hierarchical clustering of log2 FPKM values. This divided the NAC gene expression patterns into 14 clusters, indicated by numbers on the left.
(PDF)

S2 Fig Detailed subtrees of NAC subfamilies -a,b,c,e,f,g and h from the phylogenetic tree shown in Fig 2. (PDF)

S3 Fig Global phylogenetic analysis of NAC TFs. The NAC domain peptide sequences of barley (*H*. *vulg*are), common wheat (*T*. *aestivum*), *A*. *tauschii*, wild emmer (*T*. *turgidum*), wild einkorn (*T*. *urartu*) rye (*S*. *cereale*), rice (*O*. *sativa*), *B*. *distachyon*, *S*. *bicolor*, foxtail millet (*S*. *italica*), switchgrass (*P*. *virgatum*), maize (*Z*. *mays*), *Z*. *pacifica*, pineapple (*A*. *comosus*), banana (*M*. *acuminata*) and oil palm (*E*. *guineensis*) were used to construct an approximately-Maximum Likelihood tree with 1000 resamples. The NAC d-9 subgroup, was identified following Borrill et al. (2017) [7] and Pereira-Santana et al. (2015) [19]. The respective sequences are coloured in red.
(EPS)

S4 Fig Heatmap showing gene expression of wheat *Grain-NAC*s. Heatmap of gene expression values (log2 tpm values) for Grain-TaNAC and senescence-associated TaNAC orthologous genes across 25 different samples/tissue types. RNA-seq expression data are from Borrill et al.(2016) [50]. Grain-TaNACs are clustered at the bottom of the figure and mainly expressed in tissues of the developing grain S5 Fig. Alignment of Grain and senescence-associated HvNAC promoter sequences. The identical and similar conserved P-BOX2, MYBCORE, AACA motif-2, MYB1AT, RYEREPEAT, EBOXBNAPA and DPBFCORE motifs are indicated in squares.
(PDF)

S5 Fig Alignment of Grain and senescence-associated HvNAC promoter sequences. **The** identical and similar conserved P-BOX2, MYBCORE, AACA motif-2, MYB1AT, RYEREPEAT, EBOXBNAPA and DPBFCORE motifs are indicated in squares.
(PDF)

S6 Fig Cis-regulatory element annotations of wheat, rice and maize Grain-NAC promoters. The positions of seven conserved cis-elements involved in seed development are shown in wheat (A) rice (B) and maize (C) NAC promoters. Identical seed-specific motifs are represented by triangles fully coloured, while similar motifs (element with a SNP variation) are represented by triangle stroke paint. Triangles on the upper and lower side represent the orientation on positive and negative strands, respectively.
(PDF)

S7 Fig Alignment of promoter sequences from Grain-HvNAC and Grain-TaNAC homologous genes. The identical and similar conserved P-BOX2, MYBCORE, AACA motif-2, MYB1AT, RYEREPEAT, EBOXBNAPA and DPBFCORE motifs are indicated in squares.
(PDF)

S8 Fig Alignment of promoter sequences from Grain-HvNAC, Grain-OsNAC and Grain-ZmNAC homologous genes. The identical and similar conserved P-BOX2, MYBCORE, AACA motif-2, MYB1AT, RYEREPEAT, EBOXBNAPA and DPBFCORE motifs are indicated in squares.
(PDF)

S1 Table 167 barley NAC TFs identified in this study. (XLSX)

S2 Table Listing of corresponding gene IDs for the sequences used in the phylogenetic tree in Fig 2. (XLSX)

S3 Table Listing of corresponding gene IDs for the sequences used in the phylogenetic tree in Fig 4. (XLSX)

S4 Table Number of identical motifs known to be involved in seed development of both Grain- and senescence-associated HvNAC promoters. (XLSX)

S5 Table Number of identical motifs known to be involved in seed development of maize, rice and wheat Grain-NAC promoters. (XLSX)

S6 Table Number of DLN and LVFY motifs identified in NAC TFs. (XLSX)
