# Supplementary figures and images for: Genome wide characterization of barley NAC transcription factors enables the identification of grain-specific transcription factors exclusive for the Poaceae family of monocotyledonous plants
Source: PLoS One. 2018 Dec 28;13(12):e0209769. doi: 10.1371/journal.pone.0209769 (PMC6310276; doi:10.1371/journal.pone.0209769)

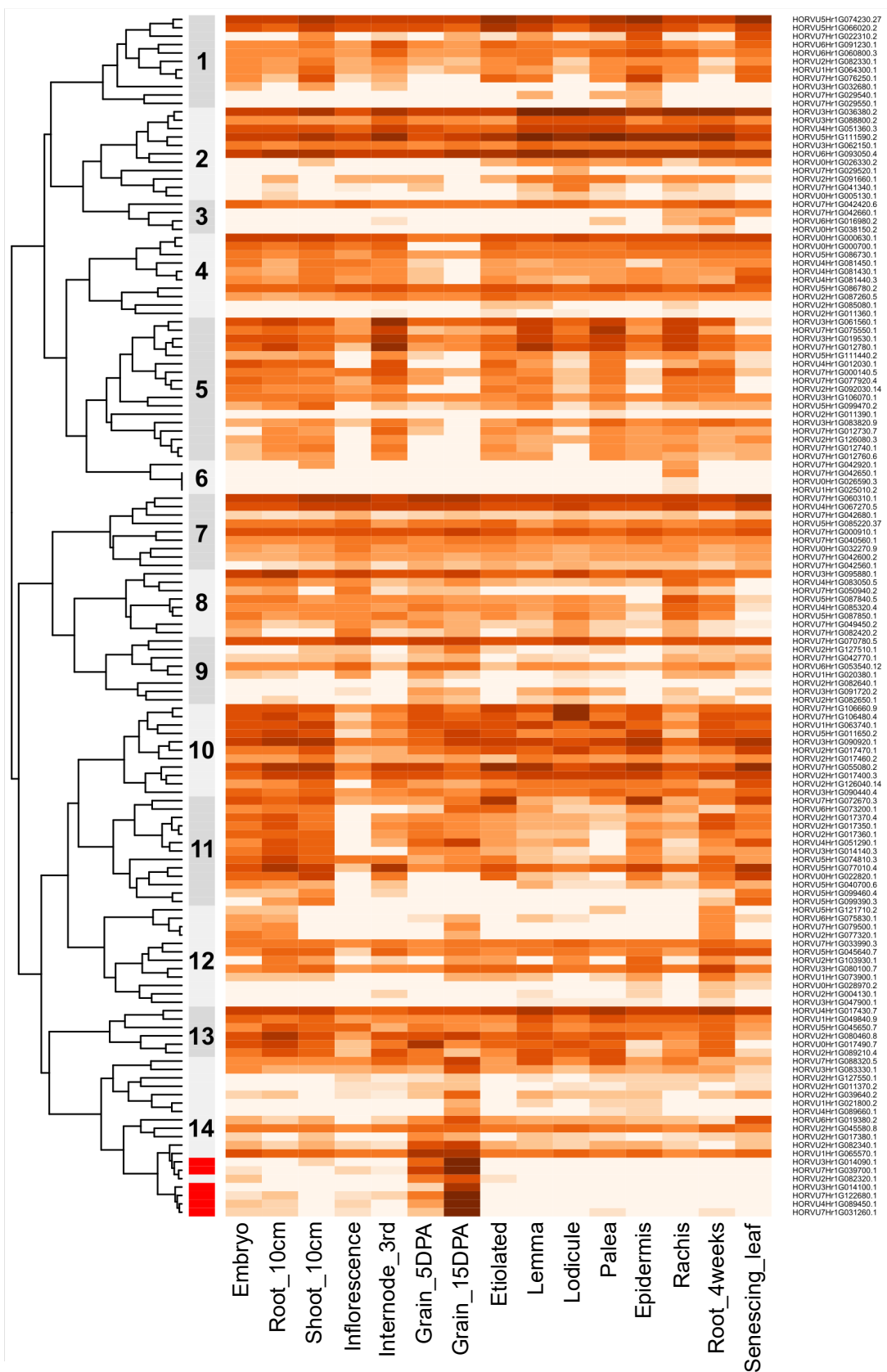

Supplement: S1 Fig — The RNA-seq expression data are taken from Mascher et al. (2017) [22], omitting one sample from the inflorescence (referred to as INF1) as this sample was characterized by very low expression levels. Grain-NACs are marked in red. A Pearson correlation distance function was used in the hierarchical clustering of log2 FPKM values. This divided the NAC gene expression patterns into 14 clusters, indicated by numbers on the left. (PDF) [file pone.0209769.s001.pdf]

NAC-a

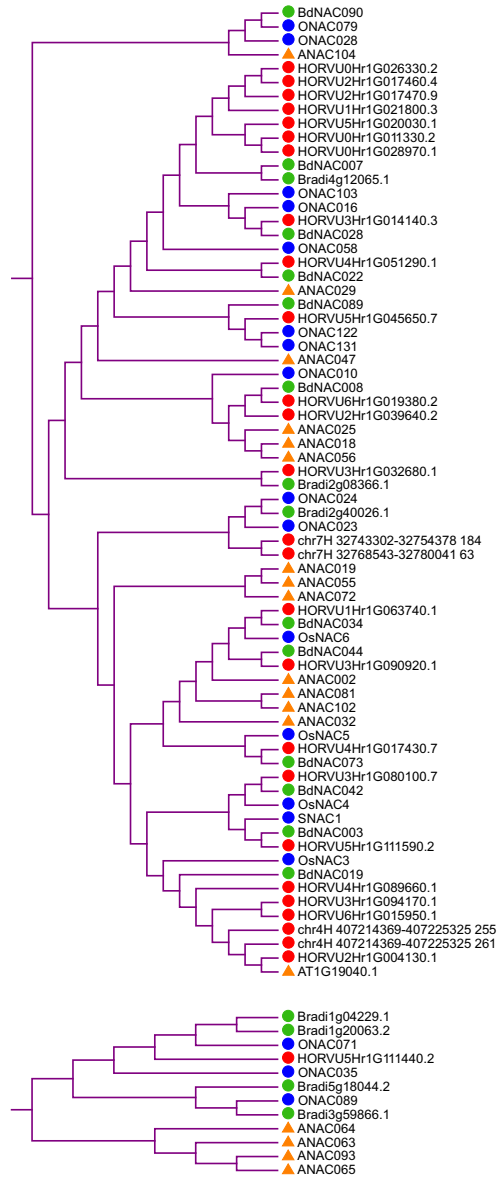

NAC-b

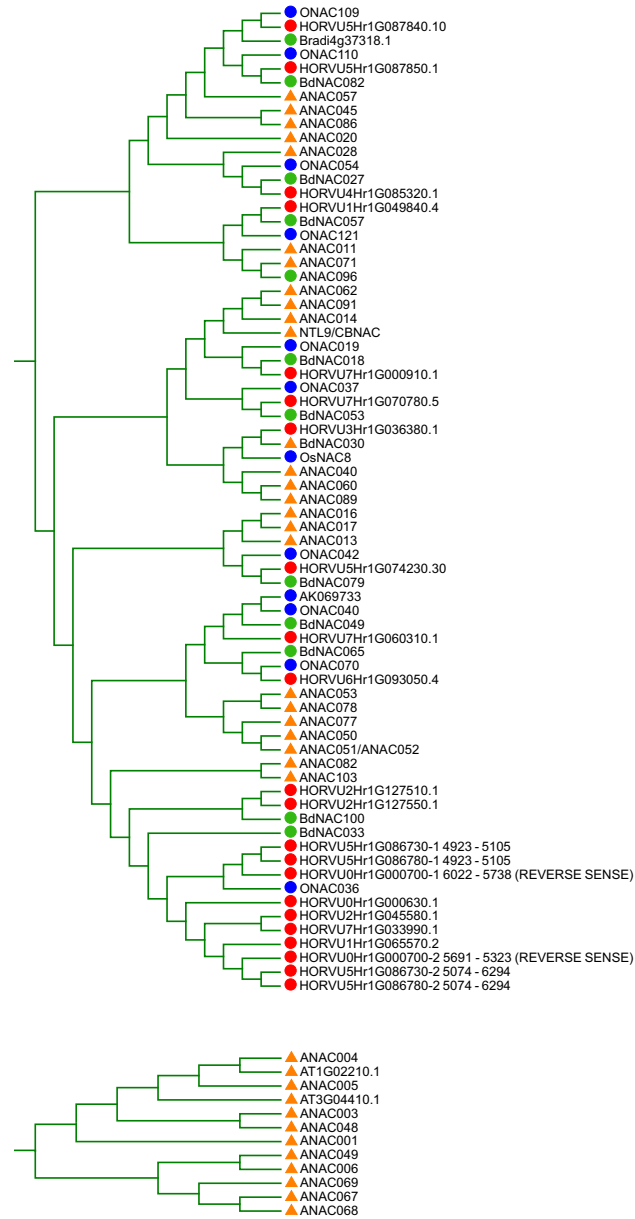

NAC-c

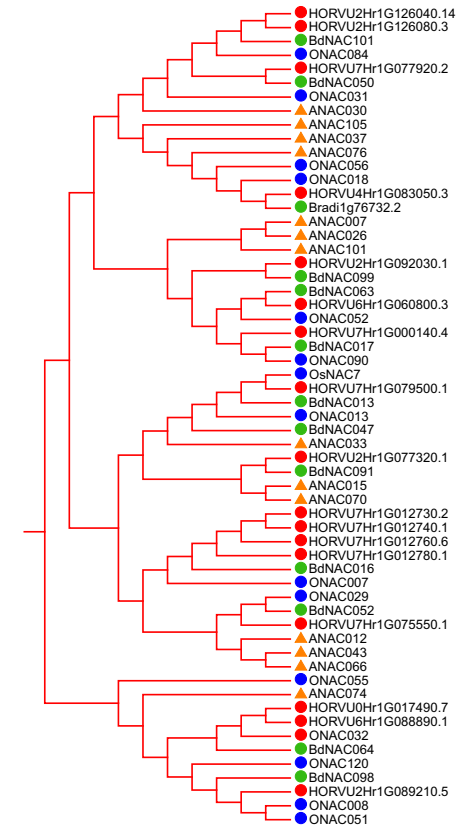

# NAC-e

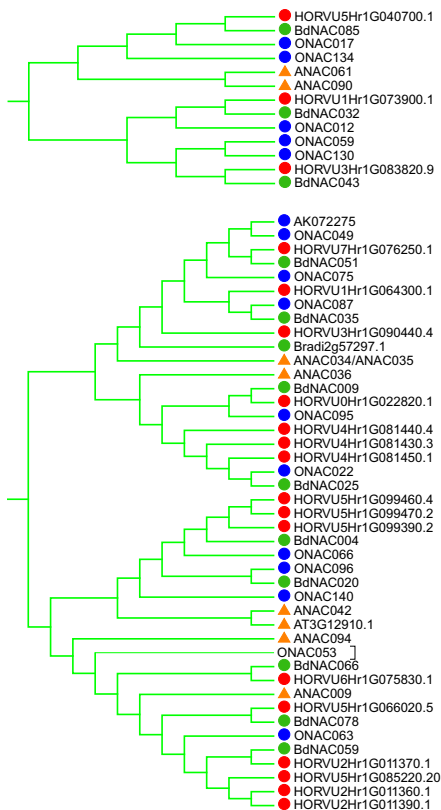

- *H. vulgare*
- *O. sativa*
- *B. distachyon*
- ▲ *A. thaliana*

# NAC-f

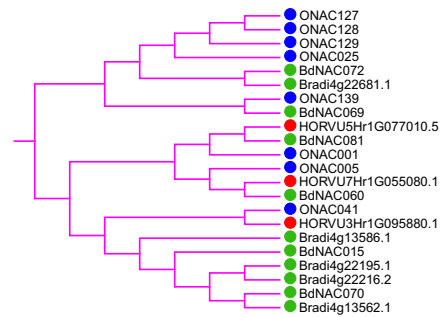

# NAC-g

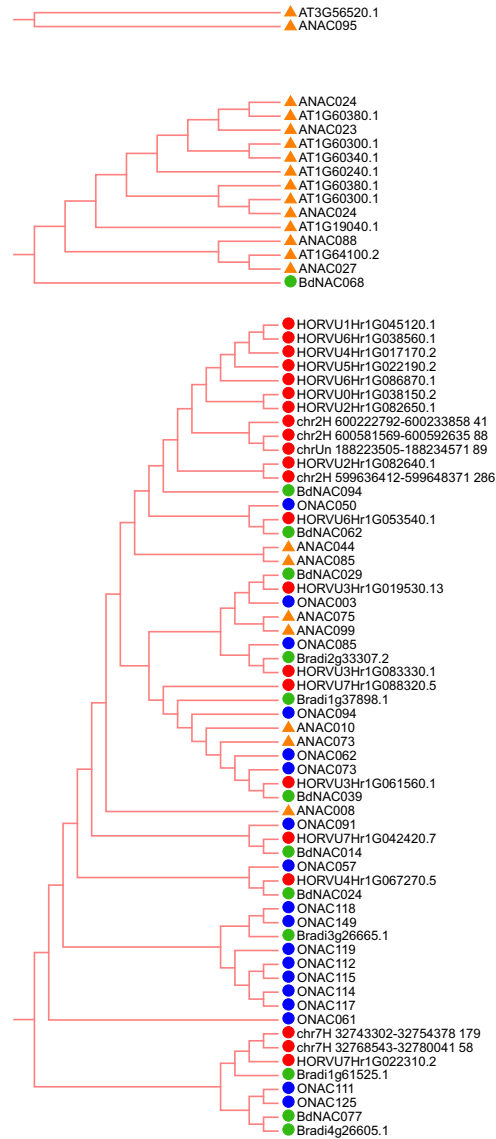

# NAC-h

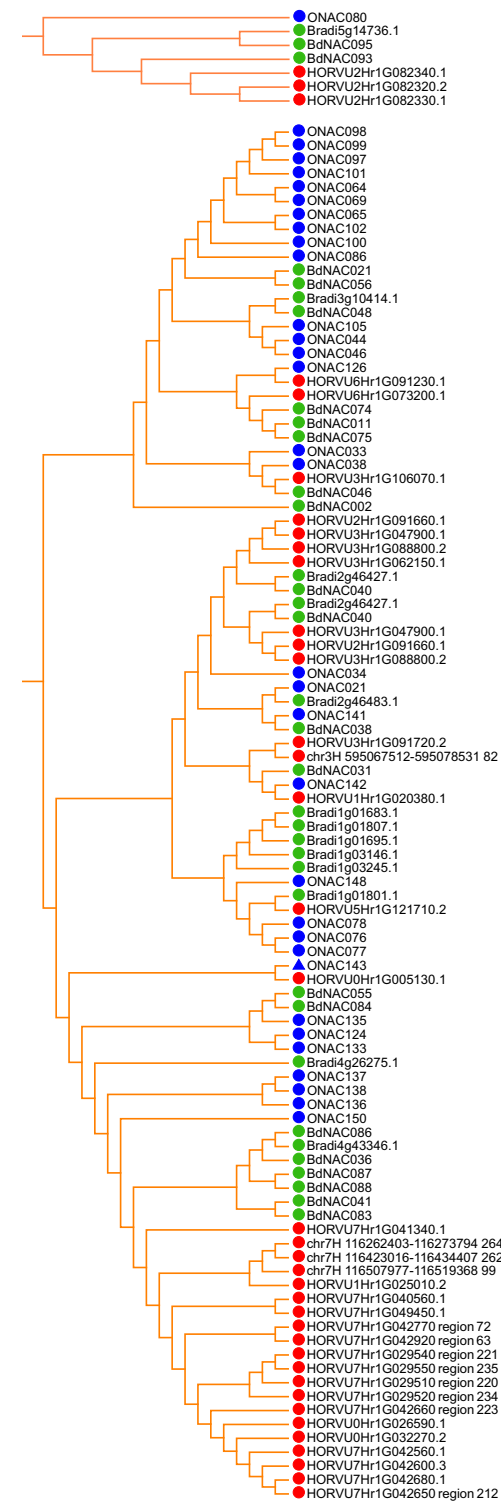

Supplement: S2 Fig — (PDF) [file pone.0209769.s002.pdf]

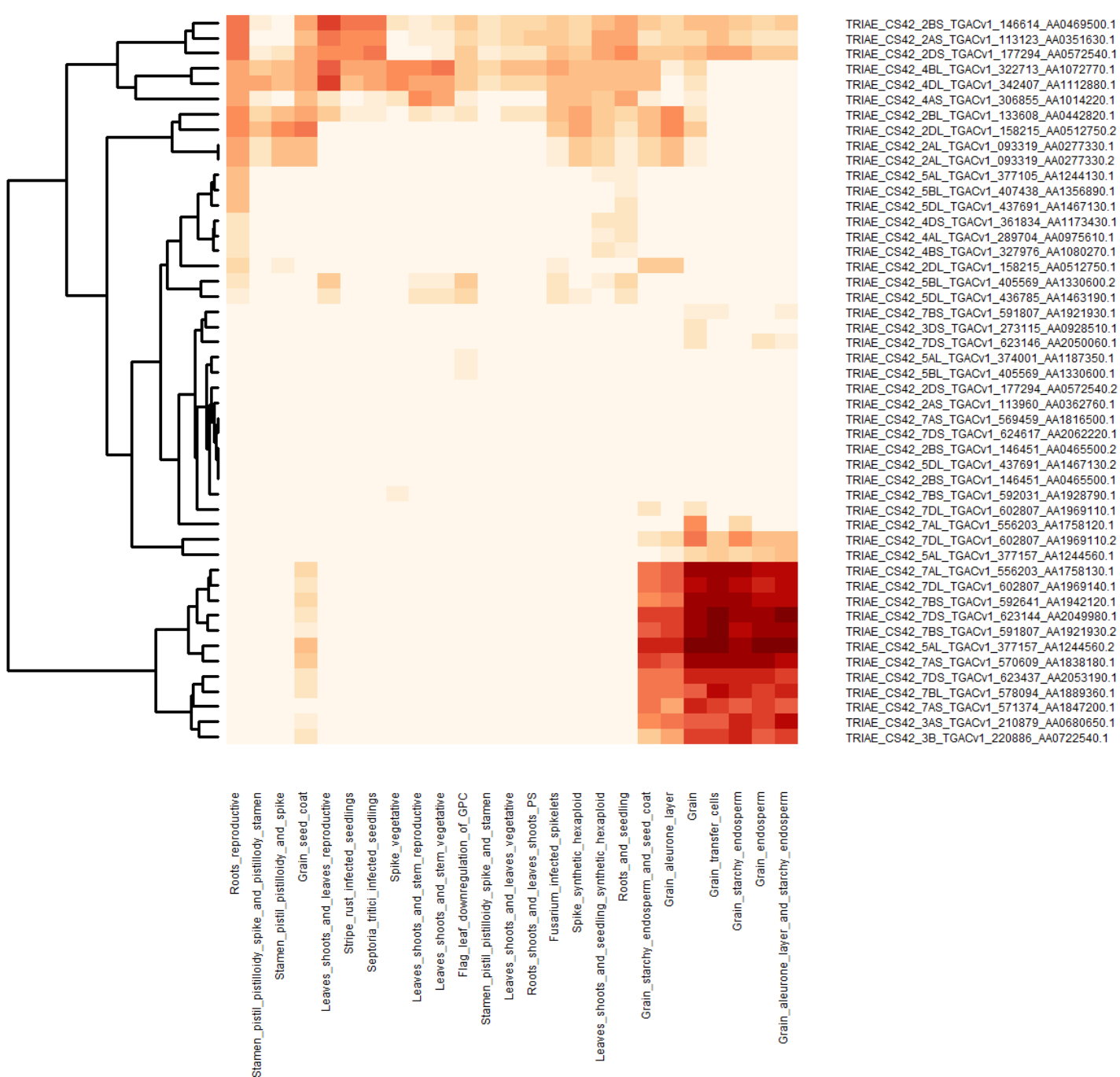

Supplement: S4 Fig — Heatmap of gene expression values (log2 tpm values) for Grain-TaNAC and senescence-associated TaNAC orthologous genes across 25 different samples/tissue types. RNA-seq expression data are from Borrill et al.(2016) [50]. Grain-TaNACs are clustered at the bottom of the figure and mainly expressed in tissues of the developing grain S5 Fig. Alignment of Grain and senescence-associated HvNAC promoter sequences. The identical and similar conserved P-BOX2, MYBCORE, AACA motif-2, MYB1AT, RYEREPEAT, EBOXBNAPA and DPBFCORE motifs are indicated in squares. (PDF) [file pone.0209769.s004.pdf]

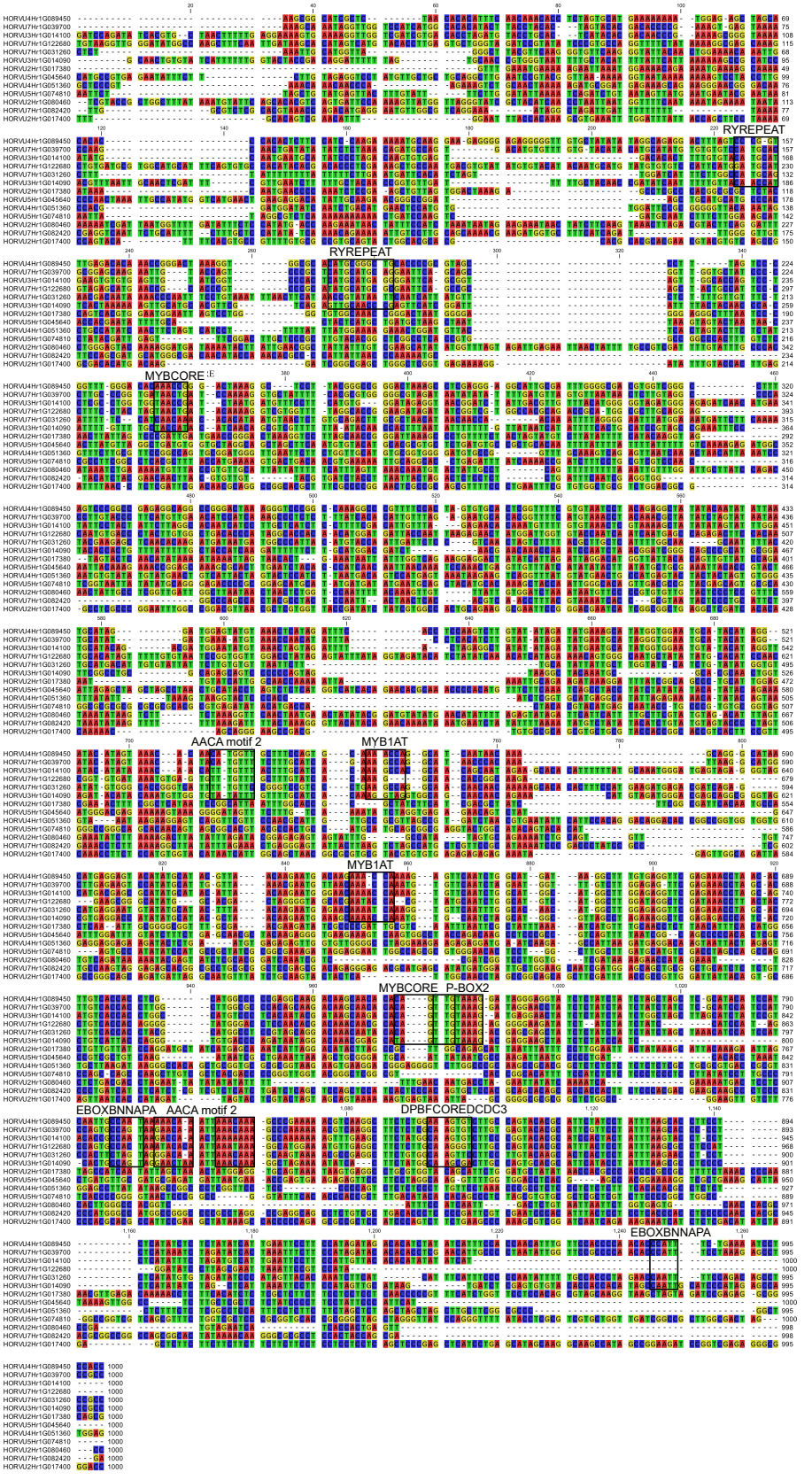

Supplement: S5 Fig — The identical and similar conserved P-BOX2, MYBCORE, AACA motif-2, MYB1AT, RYEREPEAT, EBOXBNAPA and DPBFCORE motifs are indicated in squares. (PDF) [file pone.0209769.s005.pdf]

**A***Grain-TaNAC promoters*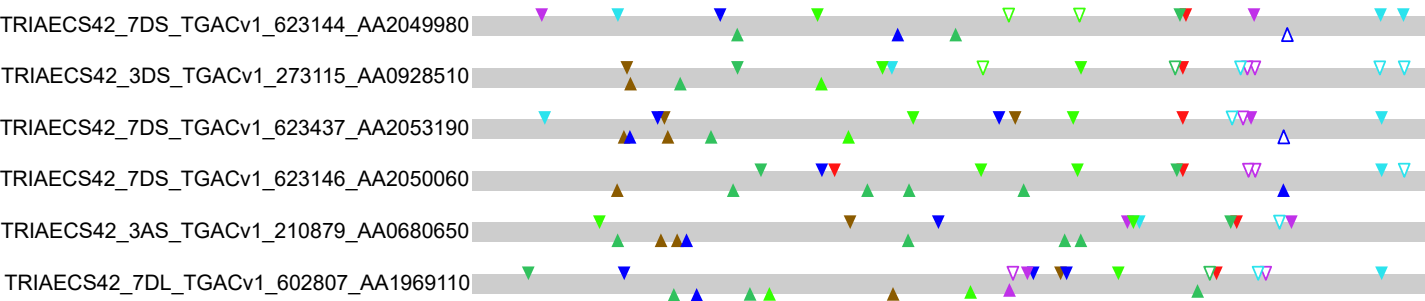**B***Grain-OsNAC promoters*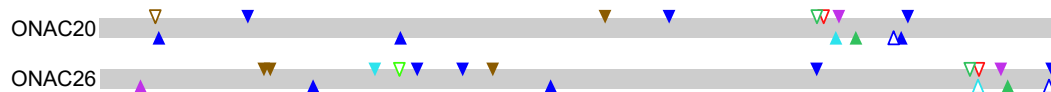**C***Grain-ZmNAC promoters*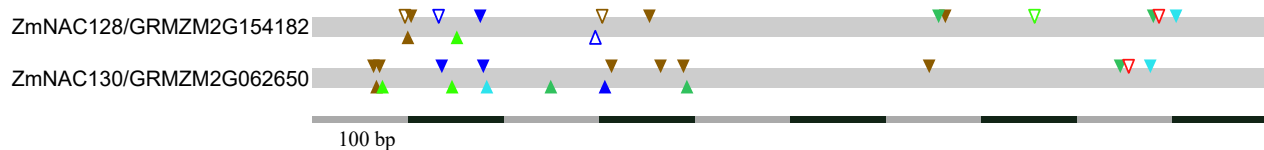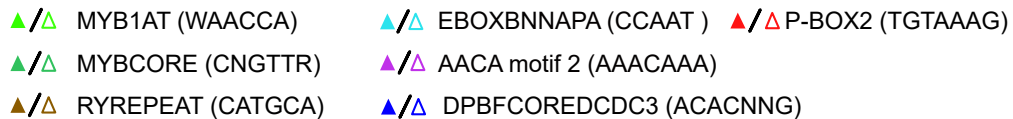

Supplement: S6 Fig — The positions of seven conserved cis-elements involved in seed development are shown in wheat (A) rice (B) and maize (C) NAC promoters. Identical seed-specific motifs are represented by triangles fully coloured, while similar motifs (element with a SNP variation) are represented by triangle stroke paint. Triangles on the upper and lower side represent the orientation on positive and negative strands, respectively. (PDF) [file pone.0209769.s006.pdf]

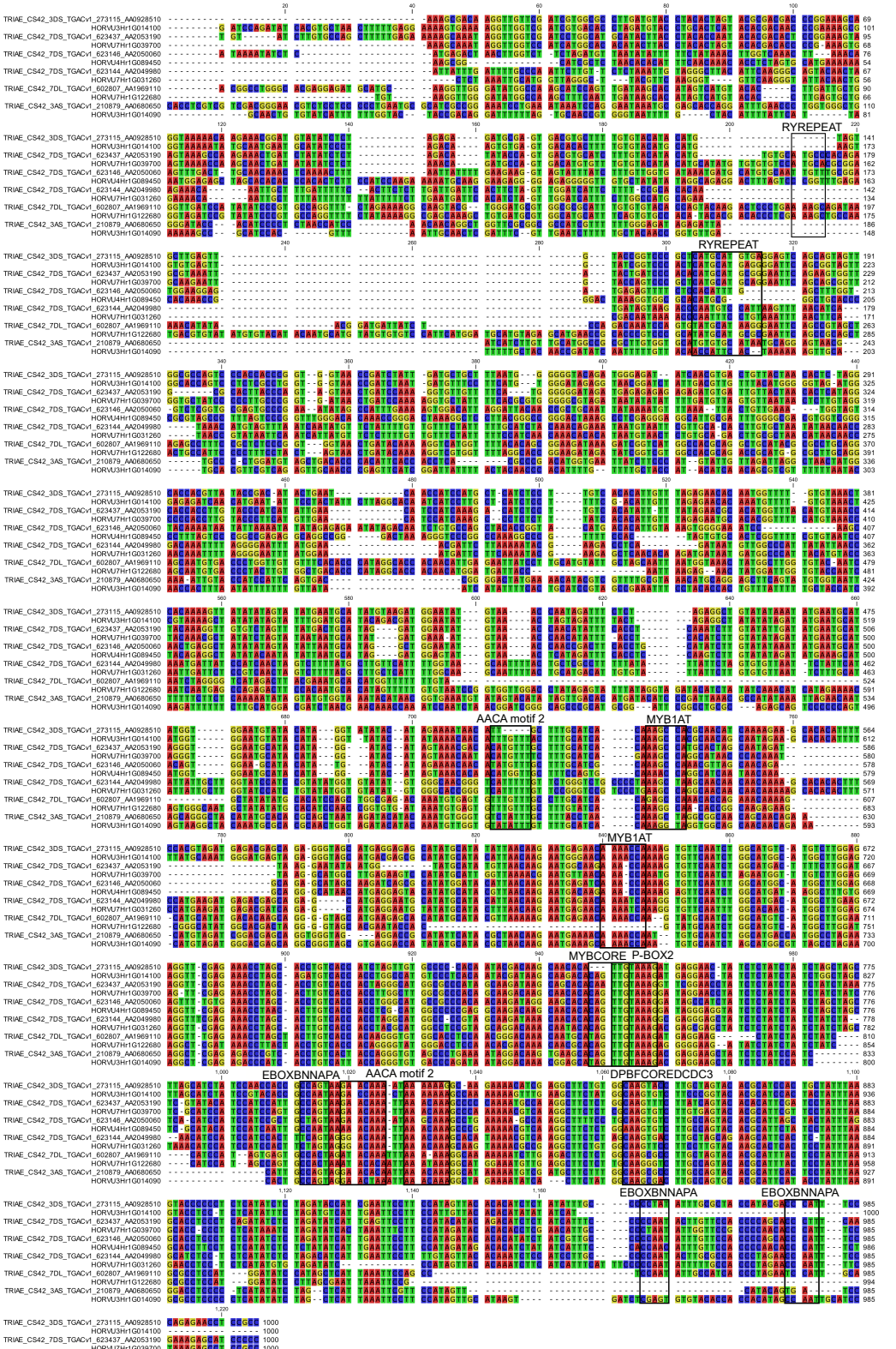

Supplement: S7 Fig — The identical and similar conserved P-BOX2, MYBCORE, AACA motif-2, MYB1AT, RYEREPEAT, EBOXBNAPA and DPBFCORE motifs are indicated in squares. (PDF) [file pone.0209769.s007.pdf]

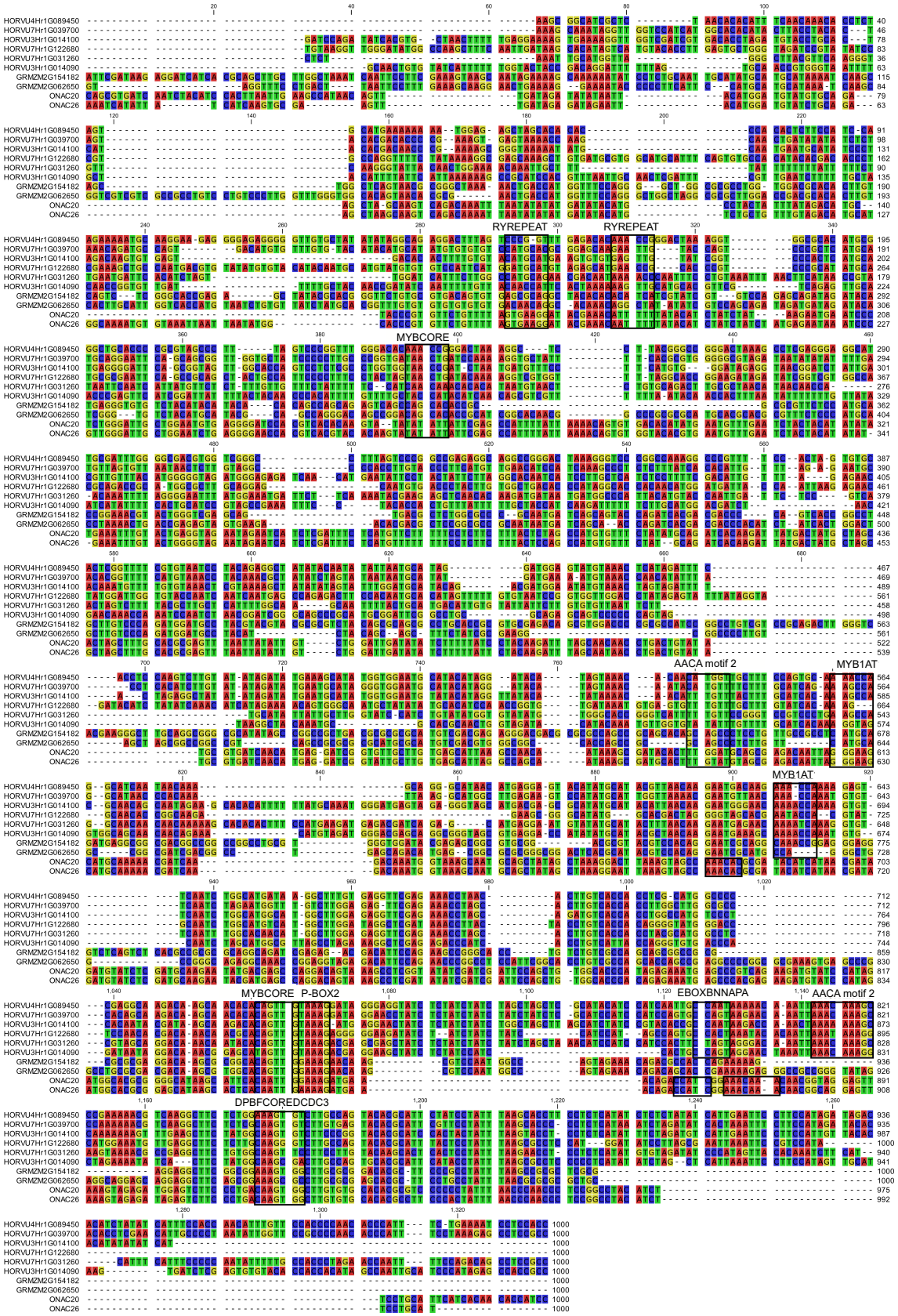

Supplement: S8 Fig — The identical and similar conserved P-BOX2, MYBCORE, AACA motif-2, MYB1AT, RYEREPEAT, EBOXBNAPA and DPBFCORE motifs are indicated in squares. (PDF) [file pone.0209769.s008.pdf]
